# A Risk of Gonadoblastoma in Familial Swyer Syndrome—A Case Report and Literature Review

**DOI:** 10.3390/jcm13030785

**Published:** 2024-01-30

**Authors:** Ewa Rudnicka, Aleksandra Jaroń, Jagoda Kruszewska, Roman Smolarczyk, Krystian Jażdżewski, Paweł Derlatka, Anna Małgorzata Kucharska

**Affiliations:** 1 Department of Gynecological Endocrinology, Medical University of Warsaw, 02-091 Warszawa, Poland; 2 Students Scientific Group of Department of Pediatrics and Endocrinology, Medical University of Warsaw, 02-091 Warszawa, Poland; 3 Students Scientific Group of Department of Gynecological Endocrinology, Medical University of Warsaw, 02-091 Warszawa, Poland; 4 Human Cancer Genetics, Biological and Chemical Research Center University of Warsaw, 02-089 Warszawa, Poland; 5 Second Department Obstetrics and Gynaecology, Medical University of Warsaw, 02-091 Warszawa, Poland; pawel.derlatka@wum.edu.pl; 6 Department of Pediatrics and Endocrinology, Medical University of Warsaw, 02-091 Warszawa, Poland

**Keywords:** familial gonadal dysgenesis, 46,XY, gonadoblastoma, Swyer syndrome

## Abstract

A complete gonadal dysgenesis (CGD) with 46,XY karyotype is known as the Swyer syndrome and belongs to the group of 46,XY differences of sex development (DSD). The main problem in patients with Swyer syndrome is the delayed puberty and primary amenorrhea. Moreover, intrabdominal dysgenetic gonads in the patient with genetic material of a Y chromosome may conduce to the development of gonadal tumors, such as gonadoblastoma or germinoma. The management of such patients is based on preventive excision of dysgenetic gonads and long-term hormonal replacement therapy. Sporadic cases are considered more common than familial cases. This paper presents two siblings with Swyer syndrome in whom gonadoblastoma was found. A thorough review of familial CGD with 46,XY DSD in the literature from the last 15 years suggests that the risk of gonadal tumors could be increased in familial compared to sporadic cases (66.6% vs. 15–45%, respectively).

## 1. Introduction

A pure or complete gonadal dysgenesis (CGD) with 46,XY karyotype is known as the Gordon Swyer syndrome and belongs to the group of 46,XY differences of sex development (DSD) [1,2]. CGD with 46,XY karyotype involves the presence of underdeveloped and undifferentiated streak gonads, which are unable to secrete any testicular hormones, such as anti-Mullerian hormone (AMH) or testosterone. This condition in the early fetal period leads to the preservation of Mullerian ducts and the development of fallopian tubes, the uterus, and upper part of vagina. Simultaneously, the lower part of the vagina is formed normally thanks to the influence of maternal and placental estrogens and lack of testosterone. Therefore, patients with Swyer syndrome are identified as females at the birth.

Typically, Swyer syndrome is diagnosed in adolescence due to primary amenorrhea or delayed puberty. Characteristic laboratory findings at the age of expected physiological puberty show hypergonadotropic hypogonadism. The cytogenetic evaluation directs the diagnosis towards DSDs because of the karyotype being discordant with the phenotypic sex in the child [3,4].

One of the most important problems associated with 46,XY DSD is the increased risk of gonadal tumors such as gonadoblastoma or germinoma. Gonadoblastoma is a rare gonadal lesion that consists of germ cells that resemble those of a granulosa or Sertoli tumors. It often develops in intraabdominal testes or in dysgenetic gonads in patients with a Y chromosome.

The *testes-specific protein Y 1 (TSPY1) gene* encoding TSPY1 protein is considered as the most significant gene, located on the Y chromosome, responsible for a high risk of tumorigenesis. This protein, physiologically produced by male spermatogonia and spermatocytes, is functioning as a protooncogenic factor when it is expressed in an incompatible niche with immature germ cells [5]. Therefore, the basic management in patients with Swyer syndrome except long-term hormonal replacement therapy is the excision of streak dysgenetic gonads to prevent the malignancy.

Swyer syndrome is a rare condition with its incidence estimated at approximately 1:80,000 to 100,000 births [4]. Usually the sporadic cases are observed, occurring singly in pedigrees. Familial cases are much more rare. Few such instances have been described in the literature [6,7,8,9,10,11,12,13,14,15,16,17,18].

In our paper, a new family with two sisters with Swyer syndrome is described and an in-depth review of familial Swyer syndrome in the literature of the last 15 years is presented (Supplementary Material File S1). In the analysis of literature data, the attention is focused on the question: whether the risk of gonadal tumors is the same in familial and sporadic cases or if it could be associated with some particular pathogenic gene variants.

A case of new familial Swyer syndrome was presented according to CARE Statement.

The study was approved by Ethics Committee of Medical University of Warsaw and patients signed informed consent.

## 2. Case Reports

### 2.1. Sister 1

Patient Information: A 17-year-old female was referred by a pediatrician to a gynecological and endocrine investigation because of a puberty disorder, including primary amenorrhea and a breast underdevelopment.

Clinical Findings: The patient was of tall stature, reaching 185 cm (>97c) and her weight was 85 kg, constituting >97c. The body mass index was calculated at 24.84 (>97c according to centile grids). The patient had female external genitalia with intact hymen and no signs of virilization. She had axillary and pubic hair concordant to the age and the female pattern (staging P3 in a Tanner scale). The breast development was B2-3 with adipose tissue. The bone age was assessed as 15 years.

Diagnostic Assessment: A serum hormone examination determined hypergonadotropic hypogonadism with FSH 71.21 mIU/mL (reference range for the follicular phase 3.03–8.08 mIU/mL) and LH 30.11 mIU/mL (reference range for the follicular phase 1.8–11.78 mIU/mL); estradiol concentration was 18 pg/dL (reference range for the follicular phase 21–251 pg/mL), 17-OH Progesterone value was 4.34 ng/mL (reference range for follicular phase 0.25–2.00 ng/mL), whereas AMH diminished: 0.15 ng/mL (normal range for women between 18–24 years: 1.62–9.0 ng/mL). Free plasma testosterone concentration was slightly above the standard for a female—0.55 µg/L (reference range: 0.14–0.53 µg/L). The rest of the serum and blood tests were within the reference range. Trans-abdominal ultrasonography visualized a small uterus (2.9 mL with 1 mm thick endometrium). A doctor experienced in adolescent pelvic ultrasonography described minor structures in the parametrium, which could be equivalents of gonads (the right gonad volume 1.4 mL, the left one 0.7 mL). Examination of peripheral lymphocytes revealed karyotype 46,XY with no additional chromosomal aberrations. The clinical picture was consistent with pure gonadal dysgenesis with 46,XY DSD, known as Swyer syndrome, and the proposal of preventive gonadectomy was discussed with the patient and the parents.

Therapeutic Intervention: The patient was referred for laparoscopic removal of the adnexa. During exploration, streak gonads were bilaterally found. An intraoperative sample of a gonad was assessed as consisting of a benign transformation. After surgery, a histopathologist confirmed the presence of dysgenetic gonads, and in the right one, the gonadoblastoma was found. The fallopian ducts were of normal structure. Immunohistochemical staining was positive for inhibin in the sex cord component, and in the case of germinal cells, it was positive for OCT4 (octamer binding transcription factor 4). CKEA3/4 staining turned out to be negative.

Follow-up and Outcomes: Serum hormones were measured 3 months after gonadectomy and testosterone concentration was 0.2 ug/L, 17-OH Progesterone decreased from 4.34 ng/mL to 0.8 ng/mL, and androstenedione from 1.93 ng/mL to 0.8 ng/mL (reference range for the follicular phase 0.34–2.63 ng/mL). These facts may indicate some hormonal activity preservation, likely having its source in the transformed sex cord component of the gonadoblastoma. After the surgery, the hormonal replacement therapy was implemented, initially with a dose of 1 mg of 17-β estradiol, and after 6 months, it was increased from 0.5 mg to 2 mg daily. After the first bleeding, additional 10 mg of dydrogesterone was administered as continuous sequential hormone therapy.

### 2.2. Sister 2

Patient Information: A 19-year-old sister of the first patient also had not been menstruating so far. After the diagnosis of Swyer syndrome in the younger sister, she was referred to the hospital for the investigation.

Clinical Findings: The patient was of tall stature (175 cm) with body mass excess (BMI 28.08) and was not sexually active. Gynecological investigation showed typical female external genitalia, breast underdevelopment (B3 in Tanner scale), and normally developed pubic and axillary hair (P4 and A3 in Tanner scale).

Diagnostic Assessment: In the hormonal profile, it was found that there were increased serum concentrations of gonadotropins, reaching, respectively, FSH 46.42 mIU/mL (reference range for the follicular phase 3.03–8.08 mIU/mL) and LH 15.06 mIU/mL (reference range for the follicular phase 1.8–11.78 mIU/mL). The serum concentration of estradiol and AMH was diminished (E2 < 10 pg/mL with normal range for the follicular phase: 21–251 pg/mL, AMH—0.31 ng/mL with normal range: 1.62–9.0 ng/mL). Plasma testosterone fit the female reference range. Additionally in the patient, a subclinical hypothyroidism of an autoimmune origin was found (TSH 9.6 uIU/mL and fT4 12.8 pmol/L, normal reference for TSH—0.35–4.94 uIU/mL, normal range for fT4 −9.01–19.05 pmol/L). Autoantibodies against thyroid peroxidase were considerably increased (anti-TPO 593.76 IU/mL, reference range 0–5.61 IU/mL), similarly to those against thyroglobulin (anti-Tg 347.57 IU/mL, reference range 0–4.11 IU/mL). Ultrasonography did not visualize gonads and determined the presence of the uterus. Cytogenetic examination revealed 46,XY karyotype. The clinical picture was consistent with Swyer syndrome.

Therapeutic Intervention: A preventive gonadectomy was performed, during which dysgenetic gonads were excised bilaterally, and in both of them, gonadoblastoma was found.

Follow-up and Outcomes: The patient needed supplementary hormonal treatment. The increasing doses of 17-ß estradiol were administered until a 2 mg dose was achieved and the first bleeding appeared. After this episode, 10 mg of dydrogesterone as continuous sequential hormone therapy was added and the patient started the regular complete hormonal therapy with estrogens and progestogen. The family denied having any other relatives diagnosed with disorders of sexual differentiation, primary or secondary amenorrhea, or infertility. Interestingly, the sisters have an unaffected brother.

In the family, both sisters, their brother, and father underwent genetic examination toward detecting mutation in *TSPY1 gene* on the Y chromosome. The procedure was performed with the use of a new-generation sequencing method (NGS). After analysis of 47.7% of the gene sequence, no pathogenic variants were detected.

## 3. Discussion

In PubMed/Medline databases of the last 15 years, 30 patients reported as familial cases of Swyer syndrome were found in whom genetic evaluation was performed [6,15,16,19,20,21,22,23,24,25,26,27] (Table 1) (method of searching shown in Supplementary Material File S1). The majority of them (27 out of 30) had undergone gonadectomy, and gonadal tumors were present in 18 out of 27 patients (66.6% of cases). The gonadal tumors were found only in patients over the age of 10 years old. In the majority of patients with familial Swyer syndrome, the scenario was similar: in the first patient, the diagnostics were initiated because of primary amenorrhea and scanty secondary sex characteristics, and the diagnosis in siblings was made when suspicious symptoms were present. In three cases, patients refused preventive gonadectomy.

Among the collected studies of 10 out of 13 families, genetic variants responsible for the development of Swyer syndrome were identified. Among these, genes *MAP3K1* and *DHH* pathological variants occurred in more than one family. Considering the risk of malignancy in gonads in patients with Swyer syndrome, the data suggest that some pathogenic variants (FTLHL17, DAX-1, DHH R124Q, del at 17q24.3) may be associated with a higher risk and some (STAR, STARD8, MAP3K1) with a lower risk of tumorigenesis.

The first report of Swyer syndrome was published in 1955 [28]. It was subsequently proven that the observed discrepancy between genetic sex and phenotype may be explained by disturbances in gonadal differentiation. Their incomplete development may be associated with pathological variants in genes that are crucial in sex differentiation. These genes include: *SRY* (*sex-determining region of Y chromosome*)*, SOX9, NR5A1, WNT4, NR0B1, DHH, GATA4, MAP3K1, WT1, FOG21, (ARX, ATRX, CBX2, DMRT1, MAMLD1, WWOX)*, FTHL17, FGF9, deletion of 9p, and duplication of Xp22 [29,30]. These genes are involved in the differentiation of primitive gonads into functioning testes. The basic genetic information that initiates this process is the sex-determining region on the Y chromosome (SRY), which in turn is responsible for regulation of the expression of key genes in differentiating testes. The key genes involved in testes development are enhanced whereas genes engaged in the differentiation of ovaries are silenced. Gonadal differentiation is dependent on activation of a signaling pathway characteristic for the following kinds of gonads: SOX9/FGF9 in testes and RSPO1/WNT4 in ovaries.

A wide spectrum of phenotypes may be observed in 46,XY DSD patients: from undervirilization and male infertility through the presence of atypical genitalia in partial gonadal dysgenesis until complete gonadal dysgenesis with a female phenotype. The latter is known as the aforementioned Swyer syndrome and is diagnosed when the gonads are completely dysgenetic and not functional, despite seemingly being normal male karyotype 46,XY. The lack of prenatal synthesis of AMH and testosterone by male fetal gonads leads to the preservation of Mullerian structures (uterus, fallopian ducts, upper part of vagina) and Wollfian duct’s atresia. In the perineal area, the transition of urogenital sinus into female external genitalia occurs due to maternal and placental estrogens. As a result, the internal and external genitalia are female. The uterus may be observed later on as normal, but it may be hypoplastic due to estrogen deficiency [31]. It is typical that these children are recognized as female at birth and are usually diagnosed at pubertal age because of lack of *gonadarche* and primary amenorrhea.

Swyer syndrome running in families is reported to be very rare. It is rather thought that most mutations arise de novo and are responsible for sporadic cases, especially those associated with the *SRY* gene [6,32,33]. Nevertheless, a prevalence of familial cases strongly suggests a genetic background of the disorder, connected with possible germinal gene disturbances rather than simple coincidence.

The most commonly reported mutations associated with Swyer syndrome, as was the case with the first one established in the 1990s, are these localized within the *SRY* gene, which plays a significant role as a testes-determining factor and initiates the process of testicular differentiation in fetus’ bipotential gonads [34]. It could be assumed that all *SRY*-related CGD are due to de novo mutations. Therefore, such a Y-linked inheritance pattern can be considered. Such a statement could be defended by assuming that not all individuals with the *SRY* gene pathogenic variants are affected. The phenotype of an individual with an *SRY* mutation is either XY-female or a typical fertile male [28]. On the other hand, an interesting possible explanation was offered by Gimelli et al. [33] and Stoppa-Vaucher [35], who suggested the presence of paternal mosaicism, probably present in the paternal germ line and not detected in peripheral lymphocytes.

In fact, an *SRY* gene mutation is found in about 15–20% of cases of 46,XY gonadal dysgenesis [34]. The rest of the putative genes could probably be localized on the autosomes and X chromosome. Among the familial presentation, there were identified CGDs due to mutations of following genes: *DHH* [7,24], *FTHL17* [20], *STARD8* [15], *SOX9* [9,16], *MAP3K1* [17], *NR5A1* [36]. Because of differences in location in the genome, various patterns of inheritance can be taken into account, such as autosomal dominant pattern (*NR5A1/SF1, DHH*—heterozygote, *WNT4* duplication, *WT1, SOX9*), autosomal recessive (*DHH* [7,24], *SF1/NR5A1* [36]), and, in the case of sexual chromosomes, the X-linked genes (*NR0B1/DAX1; ATRX, ARX, FTHL17*) [2,20].

The genetic origin confirmed by molecular evaluation in all forms of DSD reaches barely 20–30% [31]. In clinical practice, the cost-effectiveness of this procedure is questionable. However, in some cases, it could be justified. For example, the specific picture is observed in *NR5A1* mutation, which can be inherited and harbored by many family members in the pedigree, either in 46,XY or in 46,XX individuals. The clinical manifestation could be variable: from atypical external genitalia, hypospadias, and cryptorchidism in those who are genetically male, to female (46,XX) pathological gene variant carriers who are at an increased risk of developing premature ovarian insufficiency [36]. Some characteristic comorbidities or malformations, which may accompany symptoms of DSD, can help type and find the candidate genes responsible for DSD. For example, the *SOX9* mutation is associated with bone dysplasia (campomelic or acampomelic). Deletion of *9p* can manifest by CGD with psycho-motor delay [37] and *DHH* pathogenic variants are also reported in individuals with CGD and minifascial neuropathy [8,38]. *WT1* mutation is a known risk factor for DSD with early renal insufficiency and kidney tumors. Nevertheless, isolated CGDs are more often reported, including familial presentation [10,31].

A detailed collection of familial medical history may also be of great importance. Brauner et al. undertook that perspective and gathered familial data about the prevalence of hypospadias, premature ovarian insufficiency, cryptorchidism, and infertility among other members of their family [39]. Considering these factors, the percentage of “familial cases” reached 22% in their study (25/140). Among 25 patients, only data regarding two patients can be consistent with complete gonadal dysgenesis [39].

A significant problem associated with Swyer syndrome is a high prevalence of tumors within dysgenetic gonads [2,4]. Among patients without familial history, the risk of malignancy ranged between 15–45% [40,41,42] and the median age of diagnosis in these patients was 17 years (range 15–20) [43]. In our collection of familial cases, the diagnosis in siblings was earlier (average 15 years, but the youngest patient was 8 months old) and the tumors were present in 66.6% of cases.

Patients’ gonads resemble streak structures, but they contain undifferentiated gonadal tissue comprised of germ cells of delayed maturation, which express OCT4 [44]. Despite the lack of signals promoting the process of sex differentiation, other proteins encoded on the Y chromosome, such as TSPY1, may be expressed within gonadal tissue. This factor is known as testes-specific protein Y 1 and is physiologically produced by male spermatogonia and spermatocytes. It functions as a protooncogenic factor and promotes meiosis and mitosis via binding to cyclin B, thus enhancing cyclin B-CDK1 kinase activity and G2/M transition of the cells in the cycle [5]. Nevertheless, while expressed in an incompatible niche with immature germ cells, it may be conducive to the development of gonadoblastoma. Therefore, its location on the Y chromosome within a male-specific region (MSY) is also known as gonadoblastoma Y region (GBY) [45]. For the same reason, a mosaic Turner patient who possesses Y material can also be at an increased risk of gonadal tumors [46]. Moreover, the accumulation of OCT3/4-positive germ cells and loss of expression of TSPY1 has been observed by immunohistochemistry in the progression from gonadoblastoma to invasive germ cell tumors in dysgenetic gonads.

The gonadoblastoma found in our two patients is actually the most common tumor arising from dysgenetic gonads and the most frequent finding in Swyer syndrome. It was first described by Scully in 1953 [47]. As in our series, it may be located either on one side or bilaterally (the latter option occurs in approximately 40% of cases) [44]. The tumor is comprised of neoplastic cells arising from two components: sex cord stromal and germ cells, which are surrounded by gonadal stroma that sometimes contain steroid cells [44]. Such a tumor can evince hormonal activity and even be the cause of variable virilization or feminization in a patient with CGDs. In our series, the report of the first patient illustrates this possibility, as after gonadectomy with tumor removal, the concentration of androgen diminished. Similar observations were made by Moreira et al. [25].

In Swyer syndrome, the function of the *TSPY1* gene is preserved. Moreover, *TSPY1* is abundantly expressed in gonadoblastoma, but it may cease after the transformation of germinal cells into malignant germinoma [45]. Therefore, both benign and malignant lesions may coexist. In complete gonadal dysgenesis, the presence of *TSPY1* is associated with a higher risk of gonadal germ cell tumors [48]. In our report, the *TSPY1* gene in the father and affected daughters was analyzed and no pathogenic variant was found. Among gonadal tumors, a germinoma is associated with a slightly worse prognosis, since it may potentially expand locally. Nevertheless, it progresses slowly and it is rarely encountered in advanced stages, which require chemotherapeutic treatment, especially when prophylactic gonadectomy is performed, as soon as diagnosis is established, i.e., in adolescence or early adulthood. Problems may arise when patients affected with Swyer syndrome remain undiagnosed. The typical female appearance of the external genitalia does not cause any concern, until the absence of menstruation prompts seeking an explanation. If appropriate diagnostics, followed by prophylactic gonadectomy, are not undertaken in time, the risk of malignancy is high. Advanced stages of tumor were reported in the literature [4,25]. Gonadoblastoma is a premalignant lesion from which invasive germ cell tumors can develop. Excision of a gonad with gonadoblastoma prior to the development of an invasive lesion is curative. Moreover, the preventive gonadectomy of dysgenetic gonads should be bilateral, because in 40% of patients, the gonadoblastoma develops bilaterally. Some authors underline also the importance of early diagnosis and appropriate management for better QoL in patients with uro-oncological conditions [49]. Patients with Swyer syndrome are in a particularly difficult situation in this respect, because at the same time, they learn about the inconsistency of their chromosomal and phenotypic sex and the risk of malignancy. Therefore, the proper explanation of the character of disease, methods of treatment, and life perspectives is crucial to obtain the optimal compliance of the patient.

It is important to note that some tumors can produce sex hormones, estrogens as well as androgen, and this can mask the presence of gonadal dysgenesis and postpone the diagnosis, what happened in the case of the second sister in the above-described family. A familial history of Swyer syndrome can help in an earlier diagnosis of gonadal tumors that run in the family. However, sibling screening is still considered as controversial; moreover, some family members can refuse the diagnostics, as in the case reported by Moreira et al. [25]. The karyotype screening in suspicious cases among family members seems to be a gold standard. Early diagnosis is important for numerous reasons, such as in order to avoid gonadal malignancy and for proper hormonal substitution to achieve close to normal pubertal development and bone density.

Swyer syndrome remains a challenge in the event of a late diagnosis, which presents a high risk for gonadal malignancy in the affected individuals. Familial prevalence remains a rare entity, but it is in fact possible and genetic background may be even more prevalent than was previously thought.

## 4. Conclusions

A familial occurrence of Swyer syndrome is extremely rare; therefore, it is obvious that too far-reaching conclusions cannot be drawn and the data evaluation should be very careful. In patients with Swyer syndrome, it is of great importance to obtain family history about adolescent or adult female family members who still do not menstruate or have delayed puberty, so as not to overlook familial cases and to provide them with earlier diagnosis.

According to our analysis, the risk of gonadal malignancy in familial cases of Swyer syndrome seems to be increased in comparison to sporadic cases (66.6% and 15–45%, respectively). The association of malignancy and genetic background is still under consideration, but the literature search has demonstrated that particular gene variants could be linked to a higher risk of malignancy, while some genes could be protective. Pathological variants in the *MAP3K1* gene have been identified in the literature as genes associated with gonadal tumors in DSD, accounting for 13–18% of patients with 46,XY gonadal dysgenesis. The risk is additionally enhanced depending on intraabdominal gonad location. At the time of discussion about the necessity of gonadectomy, the molecular genetic evaluation could fortify the arguments for this management and improve the safety of patients with Swyer syndrome. Moreover, in complete gonadal dysgenesis, the gonads are not able to perform any hormonal functions nor are capable of reproductive cell formation. Therefore, their removal should not be considered gonadectomy in the full sense of the word—they are usually strands of connective tissue and their removal does not deprive the patient of reproductive functions, as they are not possible even if the gonads remain intact. Such a realization can be a very important argument for patients who consent to gonadectomy in the case of Swyer syndrome. Lack of consent and delayed gonadectomy may contribute to the development of more advanced tumors. It therefore seems crucial that before surgical treatment, the patient should be informed about tests confirming the lack of gonadal function, not only about the risk of malignancy.

## Figures and Tables

**Table 1:** Characteristics of patients with familial 46,XY DSD complete gonadal dysgenesis.

No.	Author	Publication Date	Nation	Familial	Number of Cases	Gene	Age of Gonadectomy	The Presence of a Gonadal Tumor yes (+) no(−)
1.	H Chen [19]	2022	China	+	2	MAP3K1 (c556A > G/p.R186G) WES, Sanger sequencing	13.5 yo	−
							14 yo	+
2.	R Tang [20]	2019	China	+	4	FTHL17 (c.GA442_443TT; p.E148L) Whole-Genome Sequencing and Variant Analysis	13 yo	+
							15 yo	+
							15 yo	+
							18 yo	+
3.	M García-Acero [21]	2019	Columbia	+	2	Duplication of DAX-1	10 yo	+
							15 yo	+
4.	E Ilaslan [15]	2018	Poland	+	2	STAR STARD8	17 yo	−
							8 mth	−
5.	M Banoth [6]	2017	India	+	3	Unidentified	17 yo	−
							22 yo	+
							29 yo	+
6.	A Granados [22]	2017	United States of America	+	4	MAP3K1 Variants: c.14_16insCGG (p.A5dup) ok.1760T>A (p.L587H) c.2291T>G (p.L764R) C. 566T>A (p.L189Q)	5 yo	−
							10 yo	−
							16 yo	−
							15 yo	−
7.	F Paris [23]	2017	France	+	1	DHH p.Trp173Cys	Lack of patient consent	
8.	R Werner [24]	2015	United States of America	+	2	DHH R124Q	17 yo	+
							30 yo	+
9.	B Bhagavath [16]	2014	United States of America	+	2	349 kb del at 17q24.3	19 yo	+
							15 yo	+
10.	A Moreira [25]	2012	Portugal	+	2	Unidentified	16 yo	+
							No detailed info	+
11.	I Filges [26]	2011	Switzerland	+	2	SRY c.347T>C	16 yo	+
							16 yo	−
12.	H Li [19]	2011	China	+	2	NR5A1 15 bp micro-duplication	21 yo	−
							Lack of patient consent	
13.	M Bergeron [27]	2010	Canada	+	2	Unidentified	11 yo	+
							12 yo	+

## Supplementary Materials

The following supporting information can be downloaded at: https://www.mdpi.com/article/10.3390/jcm13030785/s1, File S1: The methods of literature searching.
