# Supplementary material for: A Risk of Gonadoblastoma in Familial Swyer Syndrome—A Case Report and Literature Review
Source: J Clin Med. 2024 Jan 30;13(3):785. doi: 10.3390/jcm13030785 (PMC10856735; doi:10.3390/jcm13030785)

Supplementary Material File S1.

Methods of literature searching.

The systematic review was conducted and database of Pubmed/Medline was searched using the query: (familial) AND (gonadal dysgenesis) AND (46,XY). Publications from the last 15 years (1st January 2008-30th September 2023) were searched in English. The initial number of publications found was 122 items with the query: familial case presentations of complete gonadal dysgenesis with a 46,XY karyotype.

After the manual analysis of the available bibliography, we selected the publications in which the gene was identified or gonadectomy coexisted. An additional search query was added: ((familial) AND (gonadal dysgenesis) AND (46,XY)) AND ((gonadectomy) OR (genetic evaluation)). Ultimately, 13 publications were qualified [Figure S1].

Figure S1. Literature analysis adapted for PRISMA 2020 flow diagram.

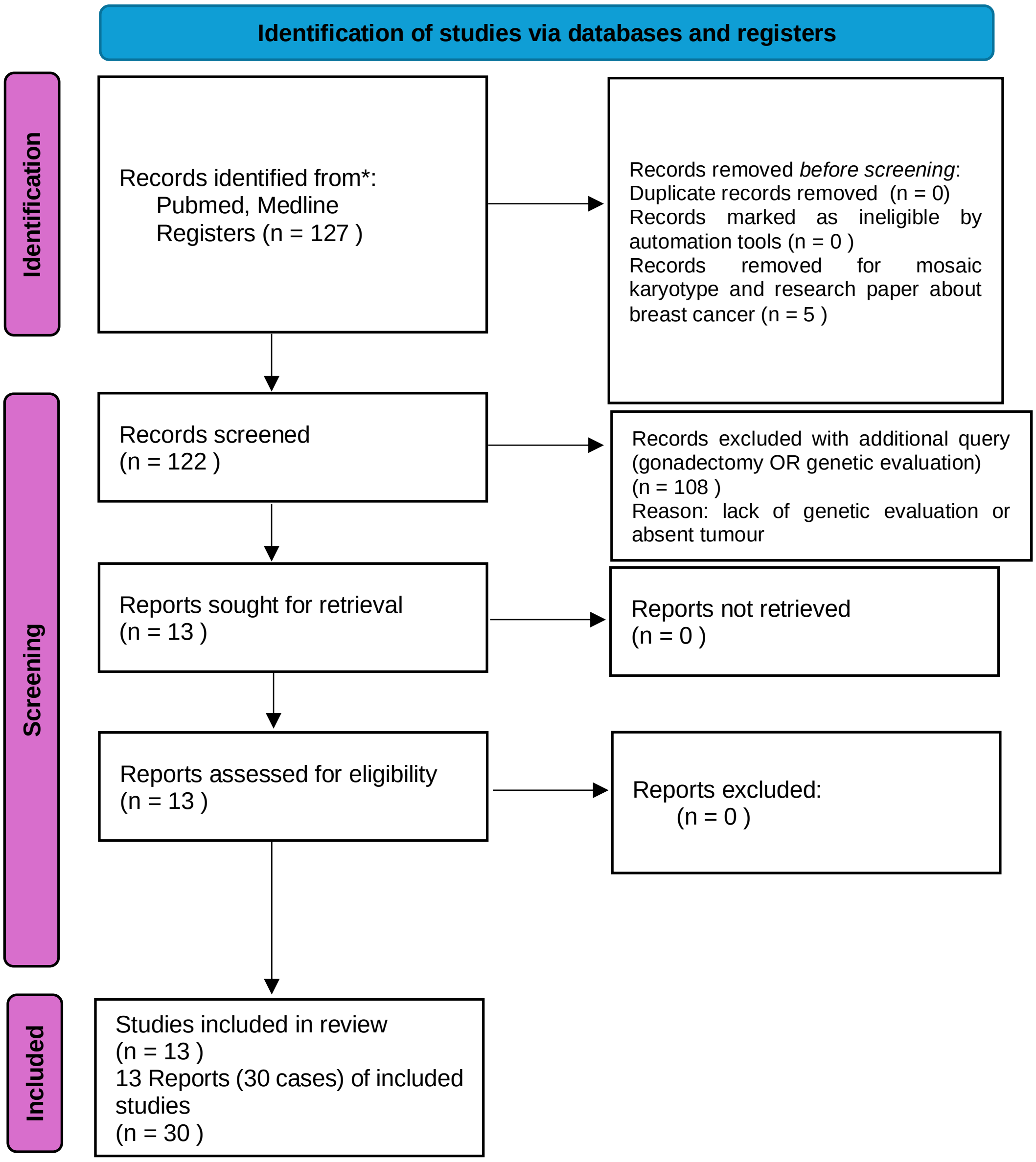

Supplement: Supplementary file 1 [file jcm-13-00785-s001.zip › jcm-2797316-supplementary.pdf]
